# Hysteresis in Lanthanide Aluminum Oxides Observed by Fast Pulse CV Measurement

**DOI:** 10.3390/ma7106965

**Published:** 2014-10-13

**Authors:** Chun Zhao, Ce Zhou Zhao, Qifeng Lu, Xiaoyi Yan, Stephen Taylor, Paul R. Chalker

**Affiliations:** 1 Department of Electrical Engineering and Electronics, University of Liverpool, Liverpool L69 3GJ, UK; E-Mails: chun.zhao@liverpool.ac.uk (C.Z.); qifeng@liverpool.ac.uk (Q.L.); xiaoyi.yan10@student.xjtlu.edu.cn (X.Y.); s.taylor@liverpool.ac.uk (S.T.); 2 Department of Electrical and Electronic Engineering, Xi’an Jiaotong-Liverpool University, Suzhou 215123, China; 3 Department of Materials Science and Engineering, University of Liverpool, Liverpool L69 3GH, UK; E-Mail: pchalker@liverpool.ac.uk

**Keywords:** high*-k* dielectrics, lanthanide aluminum oxides, pulse capacitance-voltage (CV), oxide traps

## Abstract

Oxide materials with large dielectric constants (so-called high*-k* dielectrics) have attracted much attention due to their potential use as gate dielectrics in Metal Oxide Semiconductor Field Effect Transistors (MOSFETs). A novel characterization (pulse capacitance-voltage) method was proposed in detail. The pulse capacitance-voltage technique was employed to characterize oxide traps of high*-k* dielectrics based on the Metal Oxide Semiconductor (MOS) capacitor structure. The variation of flat-band voltages of the MOS structure was observed and discussed accordingly. Some interesting trapping/detrapping results related to the lanthanide aluminum oxide traps were identified for possible application in Flash memory technology. After understanding the trapping/detrapping mechanism of the high*-k* oxides, a solid foundation was prepared for further exploration into charge-trapping non-volatile memory in the future.

## 1. Introduction

If SiO_2_ gate dielectric thickness is reduced below 1.4 nm, the resulting high gate leakage current levels due to electron tunneling effects become unacceptable for device reliability [1,2]. The decreasing sizes in complementary metal oxide semiconductor (CMOS) transistor technology required the replacement of SiO_2_ with gate dielectrics that have a high dielectric constant (high*-k*) [3,4]. The equivalent oxide thickness (EOT) is the thickness of SiO_2_ gate oxide needed to obtain the same gate capacitance as that obtained with thicker high-*k* dielectrics. Thicker equivalent oxide thickness, to reduce the leakage current of gate oxides, was obtained by introducing the high-*k* dielectric into real applications from 45 nm node technology. In recent years, various high*-k* gate dielectrics have been investigated to find suitable alternative materials [5,6,7,8,9,10,11,12]. Significant progress has been made on the screening and selection of high*-k* gate dielectrics, understanding their physical properties, and their integration into CMOS technology [13,14,15,16,17,18,19,20]. Now it is recognized that a large family of oxide-based materials emerges as candidates to replace SiO_2_ gate dielectrics in advanced CMOS applications [21,22,23,24,25,26]. Among them are cerium oxide CeO_2_ [27], cerium zirconate CeZrO_4_ [28], gadolinium oxide Gd_2_O_3_ [29], erbium oxide Er_2_O_3_ [30], neodymium oxide Nd_2_O_3_ [31], aluminum oxide Al_2_O_3_ [32], lanthanum aluminum oxide LaAlO_3_ [33], lanthanum oxide La_2_O_3_ [34], yttrium oxide Y_2_O_3_ [35], tantalum pentoxide Ta_2_O_5_ [36], titanium dioxide TiO_2_ [37], zirconium dioxide ZrO_2_ [38], lanthanum doped zirconium oxide La*_x_*Zr_1*−x*_O_2−δ_ [39], hafnium oxide HfO_2_ [40], HfO_2_-based oxides La_2_Hf_2_O_7_ [41], Ce*_x_*Hf_1*−x*_O*_2_* [42], hafnium silicate HfSi*_x_*O*_y_* [43], and rare-earth scandates LaScO_3_ [44], GdScO_3_ [45], DyScO_3_ [46], and SmScO_3_ [47]. Excellent device results have been obtained using La_2_O_3_ gate dielectric layers (*k* ~ 27), with a 3.3 nm thick La_2_O_3_ layer giving a SiO_2_ EOT as low as 0.48 nm, together with very low leakage currents [48]. However, La_2_O_3_ is chemically unstable and is easily converted to La(OH)_3_ on reaction with ambient water. LaAlO_3_ combines the advantages of the high permittivity of La_2_O_3_ with the chemical and thermal stability of Al_2_O_3_. LaAlO_3_ has a permittivity of 22–27, a large band gap (6.2 eV), and high band offsets on Si (1.8 eV for electrons and 3.2 eV for holes). Equivalent SiO_2_ thicknesses as low as 0.9–1.1 nm have therefore been achieved using LaAlO_3_. Recently, to enhance the thermal stability is the incorporation of aluminum (Al) to develop innovative multifunctional advanced lanthanide aluminates-based ceramics, NdAlO_3_ [49]. They remain amorphous up to high temperatures, leading to a large reduction in leakage current relative to poly-crystalline Nd_2_O_3_ films during CMOS processing. Therefore, the lanthanide aluminates, MAlO_3_ (M~La, Nd), are promising high-*k* materials for next generation CMOS applications.

The rapid growth of Flash memory technology has been motivated by the continuous downscaling of memory cells [50,51]. Starting from the advanced technology generation for charge-trapping Flash devices, the spacing between two adjacent gates became too narrow to arrange the metal gate to overlap the floating gate vertically in minimum feature-sized standard cells [52,53]. The introduction of high*-k* materials into floating-gate Flash memory has been proposed as a potential solution [54,55,56]. A fundamental understanding of the trap mechanism in new dielectric materials was critical for write/erase, retention and endurance properties of Flash memory [57,58,59]. Therefore, new ideas and approaches are required. These include: Negative-Bias Temperature Instability (NBTI), NBTI lifetime prediction, fast reliability screening and charge pumping techniques. These measurement techniques are required to understand of the underlying science of these dielectrics. Reliability degradation, defect loss, slowdown, and device lifetime enhancement, energy and spatial distribution, electron trapping and interface states, time-dependent defect variation were actively investigated for the high-*k* dielectrics for several years.

Prior to this paper, many novel measurement approaches were proposed for the next generation Flash memory since 2005 [60,61,62,63,64,65,66,67,68]. Various significant findings related to traps within the gate oxides were also reported [69,70,71,72]. In this paper, a novel electrical characterization of Metal Oxide Semiconductor (MOS) capacitance: pulse capacitance-voltage (CV), was proposed in detail. The accuracy and reliability of the pulse CV testing system was comprehensively verified and examined, and shown to be a powerful measurement method for dielectric characterization of high*-k* materials. Large hysteresis effects caused by oxide traps were observed in lanthanide aluminum oxides, which was critical for the development of the charge-trapping Flash memory in the future.

## 2. Experimental Section

High*-k* dielectrics, LaAlO_3_, NdAlO_3_ thin films, were deposited on *n*-type Si (100) substrates using liquid injection atomic layer deposition (ALD), carried out on an Aixtron AIX 200FE AVD reactor (Aixtron, Herzogenrath, Germany) fitted with the “Trijet”™ liquid injector system. The LaAlO_3_ thin films grown by ALD are all La deficient, with the La:Al ratio varying from 0.50 to 0.61 over the growth temperature range of 160–300 °C. As gas phase reactions are absent in ALD, the precursor is likely to remain intact until reaching the growth surface. It is therefore unsurprising that there is little variation in the La:Al ratio, although the reason for the La deficiency in the thin films is not known. The thickness of the LaAlO_3_ thin film grown by ALD is 28 nm. Near stoichiometric NdAlO_3_ thin films were grown by utilizing the single-source precursor [NdAl(OPr*^i^*)_6_(Pr*_i_*OH)]_2_. Selected thin films were subjected to high-temperature (750–950 °C) post-deposition annealing (PDA) in pure nitrogen (N_2_) ambient for 60 s. Subsequently, a post-metallization forming gas anneal (FGA) was carried out at 400 °C for 30 min using H_2_:N_2_ in the ratio 1:9, together with a control as-deposited sample. The high-*k* thickness and a thin native oxide interlayer, adjacent to the silicon substrate, changed from 11 and 1.5 nm, respectively, to 10.4 and 2.5 nm, respectively, after 950 °C PDA. This could be due to inter-diffusion of oxygen between SiO_2_ and NdAlO_3_. Here, NdAlO_3_ thin films samples discussed in the manuscript was after PDA. A thermal SiO_2_ sample was grown using dry oxidation at 1100 °C to provide a comparison with the high*-k* stacks. MOS capacitors were fabricated by thermal evaporation of Au gates through a shadow mask with an effective area of 4.9 × 10^−4^ cm^2^. The backside contact of selected Si wafers was cleaned with a buffer HF solution and subsequently a 200 nm thickness of Al film was deposited on it by thermal evaporation.

**Figure 1:**
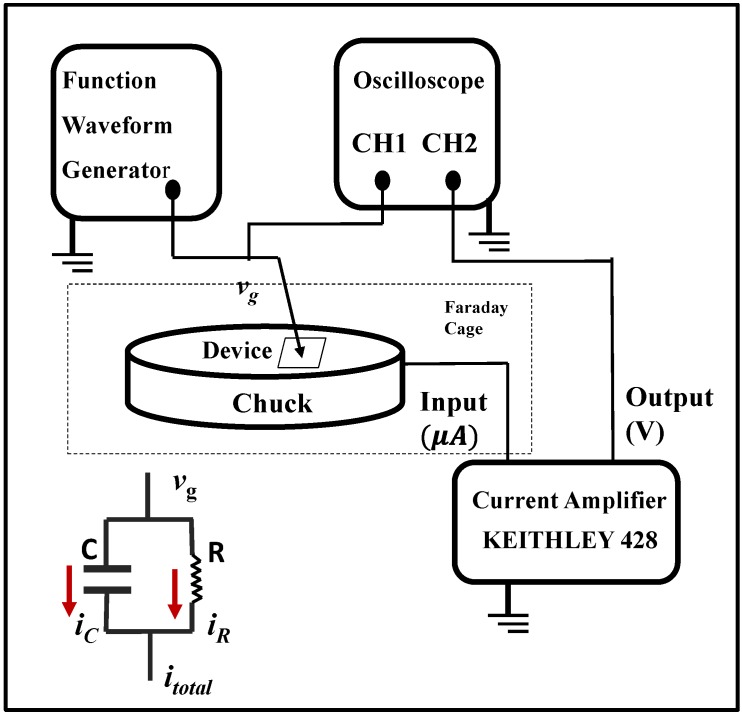
Pulse capacitance-voltage (CV) measurement system structure chart. A functional/arbitrary waveform generator was implemented to generate the voltage pulse waveform. The current through the under-testing device was fed into a current amplifier and then was amplified as an output voltage signal. An oscilloscope was used to monitor the input and output voltage signals.

The pulse CV measurement system was developed and implemented to probe the MOS capacitor sample with high*-k* thin film, and its system structure chart is shown in Figure 1. We used a functional/arbitrary waveform generator to input a pulse voltage waveform (*v_g_*) for the sample. The related current through the sample (*i*_total_) was fed into a current amplifier and then was amplified as an output voltage signal (*v*_CH2_). Channel two of the oscilloscope was used to track the output voltage, while the input pulse voltage waveform was monitored in channel one. There was no specific requirement for the oscilloscope model. Here, the DG1302 oscilloscope was employed. In terms of functional/arbitrary waveform generator, DG3061 (RIGOL, Beijing, China)/DG2041 (RIGOL)/HP8110 (Agilent, Santa Clara, CA, USA) could be implemented to produce pulse voltage. The Keithley 428 current amplifier (Keithley, Cleveland, OH, USA) was selected for the pulse CV measurement system. The symbol diagram of an under-testing device is also modeled in the inset of Figure 1. For MOS capacitor samples with the high-*k* thin film, we could make it simple as the parallel combination of a capacitor (C) and a resistor (R). Accordingly, the current flowing through the capacitor and resistor are *i_C_* and *i_R_*, respectively. Details are discussed in the following section.

## 3. Results and Discussion

In order to verify the working principle behind the pulse CV measurement system, firstly, a discrete commercial ceramic capacitor with a near infinite resistance value was used to replace the MOS capacitor sample shown in Figure 1. The value of the capacitance component was below 1000 pF. Via the relation between voltage and current, the following formula is derived:

(1) $$i_{C}\left(t\right)=\frac{v_{\text{CH}2}\left(t\right)}{A}=C\cdot \frac{dv_{g}\left(t\right)}{dt}$$

where *v*_CH2_ is the output voltage of the current amplifier (recoded by channel two of the oscilloscope). *v_g_* is the input voltage of the sample. *A* is an amplification factor of the current amplifier and *C* is the capacitance value of the device under test. Re-arranging, *v*_CH2_ could be obtained:

(2) $$v_{\text{CH}2}\left(t\right)=A\cdot C\cdot \frac{dv_{g}\left(t\right)}{dt}$$

**Figure 2:**
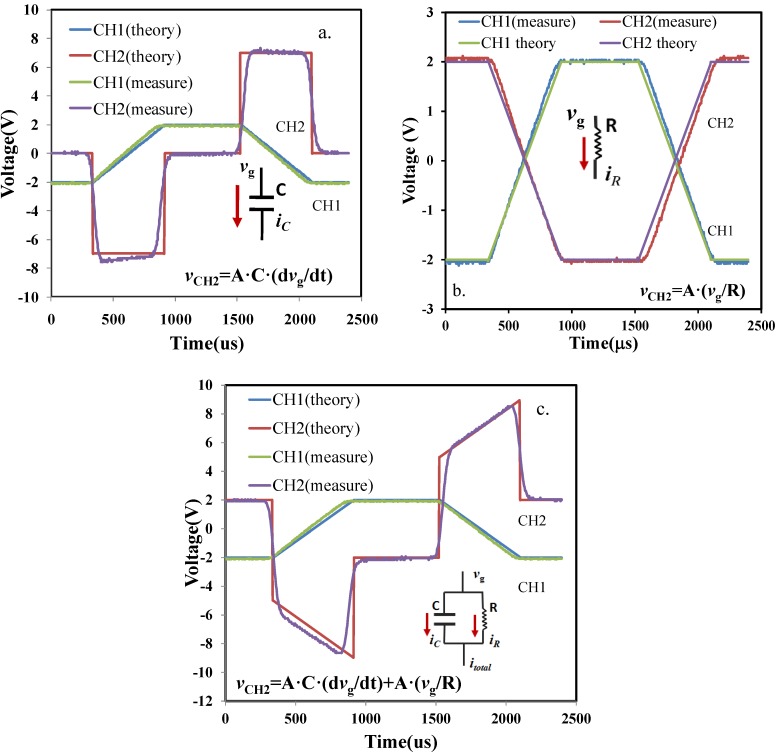
(**a**) Voltage (V) *versus* time (μs) of a discrete capacitor. (**b**) Voltage (V) *versus* time (μs) of a discrete resistor. (**c**) Voltage (V) *versus* time (μs) of a discrete capacitance parallel with a discrete resistance. CH1 denotes the input voltage waveform (*v_g_*), while CH2 represents the output voltage waveform (*v*_CH2_). Theory curves were presented based on formula calculation. Measurement data were recorded by channel 1 and channel 2 of the oscilloscope.

Based on the formula above, the theoretical *v*_CH2_ could be calculated as a reference to compare the measurement results. In Figure 2a, the measurement results from channel one (green) and channel two (purple) of the oscilloscope are presented. Following the derived formula, we plotted the theory data for channel one (blue) and channel two (red). Obviously, it was observed that the theory data fitted well with measurement results.

A discrete resistor with a resistance value *R* as the under testing device to replace the discrete commercial ceramic capacitor was then investigated. The current flowing through the resistor was given:

(3) $$i_{R}\left(t\right)=\frac{v_{\text{CH}2}\left(t\right)}{A}=\frac{v_{g}\left(t\right)}{R}$$

(4) $$v_{\text{CH}2}\left(t\right)=A\cdot \frac{v_{g\left(t\right)}}{R}$$

The comparison between theory and measurement is demonstrated in Figure 2b. Like the result of the discrete capacitance, we found the theory and measurement had an impressive consistency.

Finally, the parallel combination of the discrete capacitor and the discrete resistor was merged. The related formula was updated as:

(5) $$i_{\text{total}}\left(t\right)=\frac{v_{\text{CH}2}\left(t\right)}{A}=i_{C}\left(t\right)+i_{R}\left(t\right)=C\cdot \frac{dv_{g}\left(t\right)}{dt}+\frac{v_{g}\left(t\right)}{R}$$

(6) $$v_{\text{CH}2}\left(t\right)=A\cdot C\cdot \frac{dv_{g}\left(t\right)}{dt}+\;A\cdot \frac{v_{g}\left(t\right)}{R}$$

In Figure 2c, it was found that the theoretical calculation agreed well with measurement data. In summary, the working principle in terms of the pulse CV measurement system is mainly attributed to the current flowing through the device under test. In this paper, the measurement of MOS capacitance with parasitic resistance (usually the value was above 2 MΩ) was the key issue.

**Figure 3:**
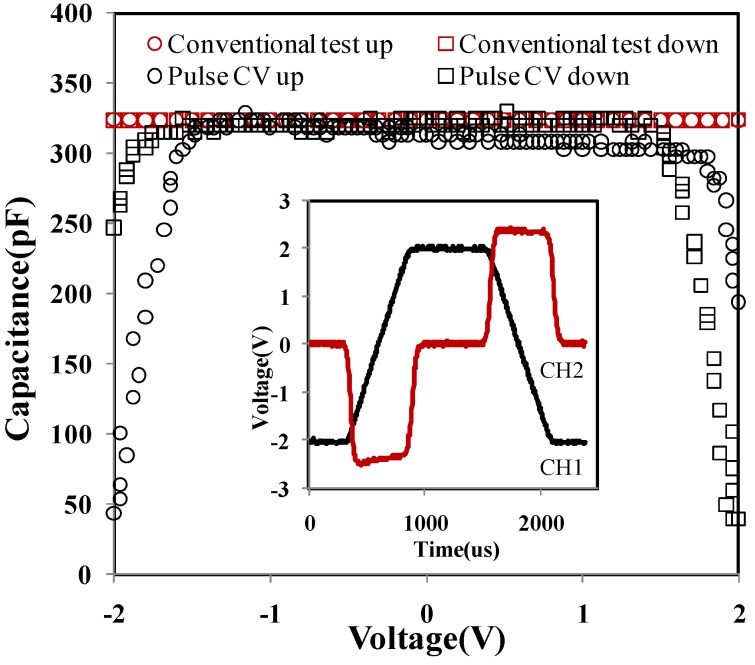
Capacitance (pF) *versus* voltage (V) of a discrete capacitor. Inset: Voltage (V) *versus* time (μs) of a discrete capacitor using the pulse CV measurement. CH2 was used to extract the capacitance value. Conventional test up and pulse CV up: *v_g_* forward from −2 to +2 V (the up trace). Conventional test down and pulse CV down: *v_g_* backward from +2 to −2 V (the down trace).

Before considering a MOS capacitance, the extracted capacitance data from a discrete capacitor should be fully understood. As an example, we tested a 330 pF discrete capacitor. From Equation (2) and an inset in Figure 3, the extracted CV graphs are shown in Figure 3, where we place the original voltage-time (VT) graphs as the inset. Pulse CV up/down (black) means that the CV results were extracted from the rise/fall edge of the input pulse on channel 1 in the inset. Before utilizing the pulse CV technique, a conventional CV test was commonly carried out via Agilent4275/4284 LCR meter (Agilent, Santa Clara, CA, USA). The related CV results (red) are presented in Figure 3. In the conventional test, up/down means the measurement starting from low/high to high/low voltage. The comparison was also made between the conventional test and the pulse CV measurement as shown in Figure 3. Besides high accuracy superposition, the CV curves from the pulse CV measurement need a rise to reach the true capacitance value within the voltage range from −2 to −1.5 V. Similarly, a fall from the true capacitance value was also observed within the range of 1.5–2 V. The phenomenon was mainly due to the response time of the oscilloscope and the capacitance charging and discharging issues. In consequence, the middle part of the full CV curves was preferred, which reflected the actual capacitance value of the discrete capacitor, like −1.5–1.5 V within the range of −2–2 V.

**Figure 4:**
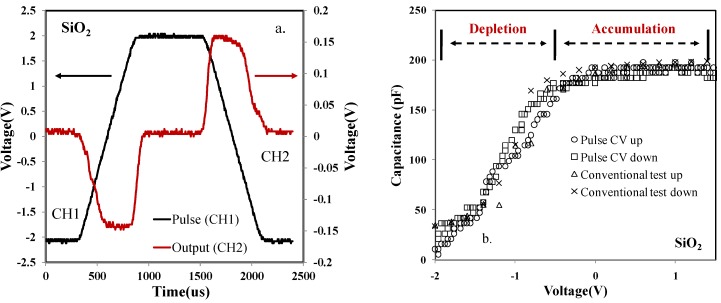
(**a**) Voltage (V) *versus* time (μs) of a thermal SiO_2_ MOS sample. (**b**) Capacitance (pF) *versus* voltage (V) of the SiO_2_ MOS sample.

After the comparison between the conventional and pulse CV test for a discrete commercial ceramic capacitor, a thermal oxide (SiO_2_) MOS capacitor sample was investigated. Figure 4a shows the original VT data from both channel one and channel two of the oscilloscope. Calculated from the Equation (6), the related CV results are given in Figure 4b. Similarly, a conventional CV test was employed to compare with the new pulse CV technique. From Figure 4b, vertically the pulse CV and conventional CV results matched consistently. There was no variation either in accumulation or depletion region. Furthermore, horizontally there was no clear shift for the pulse and conventional CV test. This is because there were no significant traps located in the oxide layer of the thermal SiO_2_ MOS system. Therefore, it should be the case that no flat-band voltage shift was observed. In other words, the fast (pulse CV) and slow (conventional test) CV measurements were expected to be the same, which was supported by our finding shown in Figure 4b. It can be concluded that the pulse CV system is suitable for MOS capacitance measurement. This provides a convincing basis for investigation of more complicated MOS capacitors with high*-k* thin films.

**Figure 5:**
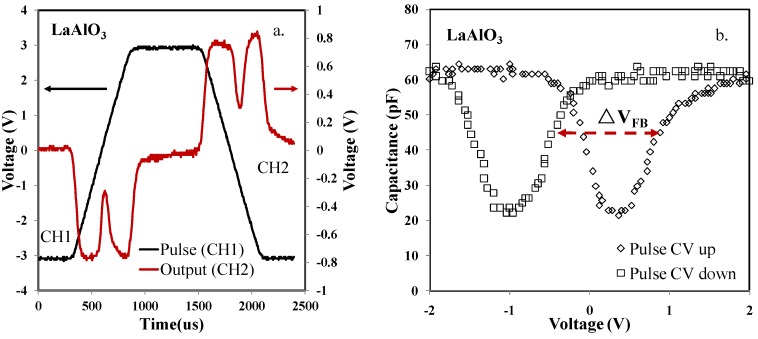
(**a**) Voltage (V) *versus* time (μs) of A LaAlO_3_ sample. (**b**) Capacitance (pF) *versus* voltage (V) of the LaAlO_3_ sample. In the pulse CV test, considerable flat-band voltage shift was observed.

The high*-k* thin film of LaAlO_3_ was now under research. The MOS capacitor samples with LaAlO_3_ were probed using pulse CV technique. The VT results are shown in Figure 5a. Unlike the discrete capacitor and the SiO_2_ MOS capacitors introduced before, there were two distinct peaks in channel two in the up (forward from −3 to +3 V) and down (backward from +3 to −3 V) trace of the input pulse, respectively. When we did the extraction based on Equation (6) into CV results in Figure 5b, the distinct peaks indicated that a strong inversion occurred when frequency is low. The capacitance density in the accumulation region was 1.27 × 10^5^ pF·cm^−2^, when the electric field within the oxide is 7.14 × 10^7^ V·m^−1^. In the inversion region, an inversion layer exists at the silicon surface. In response to the low frequency analog continuous (AC) signal, inversion layer charges can be supplied and removed quickly enough to respond to changes with the gate AC signal voltage, and incremental charge is effectively added or subtracted at the surface of substrate. Most importantly, it was found that a clear horizontal voltage shift happened between the up and down trace. The shift was around 1.3 V. However, only a horizontal voltage shift of 0.4 V was observed in the same samples from the conventional CV test. It was proved that the pulse CV technique was more accurate to track traps in oxides, avoiding trapped electrons/holes recovering/detrapping within the testing time interval of the conventional CV test. The obvious horizontal voltage shift was induced by trapping and detrapping of electrons/holes between the up and down trace of channel one in Figure 5a. When the positive voltage (+3 V) is forced on the metal side, net positive charges induce a negative shift of the C-V curve. While, if a negative voltage (−3 V) is applied on the metal side, net negative charges cause a positive shift of the C-V curve. As a discussion, a possible explanation is that the shifts might be related to as-grown positive charges and as-grown electron traps. It was reported that there are as-grown fixed positive charges (oxygen vacancies) and as-grown electron traps in high-*k* oxides [57,73,74,75]. If both as-grown positive charges and as-grown electron traps in high-*k* oxides have a high energy level, when *V_g_* < 0, the as-grown positive charges are compensated by electrons, which come from the metal gate and trapped into the as-grown traps; while when *V_g_* > 0, electrons are detrapped to the metal gate through tunneling, which causes that (1) the net charges in the oxide are positive and then (2) the CV curve negatively shifts.

**Figure 6:**
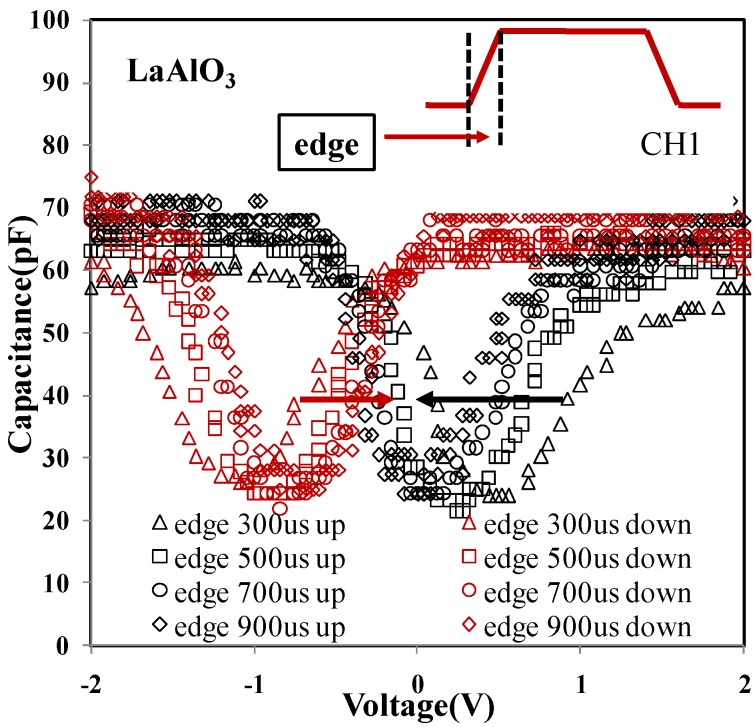
Capacitance (pF) *versus* voltage (V) of a LaAlO_3_ sample. The variation of flat-band voltage was decreased with increasing pulse edge time.

The impact of testing time on the horizontal shift of the CV curve was considered. In the pulse CV technique, edge time for both rise and fall of the voltage pulse was critical to estimate the trap density located in the oxide. The single pulse CV technique described in the paper can be used to accurately measure the charging-induced flat-band voltage shift. Furthermore, a two-pulse CV technique was developed to measure the flat-band voltage shift caused by discharging the traps in dielectrics [63]. The rising and falling slopes of a pulse signal applied to the gate will give rise to a displacement current proportional to the capacitance and the pulse ramp rate (see Equation (1)). Less edge time means less test time among each voltage biases. The rise edge time is shown in the inset of Figure 6. When the edge time was increased to a relatively large value (~10 s), the test process of the pulse CV was equal to the conventional CV test carried out via a LCR meter. In Figure 6, it was observed that the flat-band voltage shift was narrowed with increasing edge time and it indicates that the flat-band voltage shift is partially recovered during the edge time of the pulse. Trapped electrons/holes recovering were sensitive to time. Therefore, the time-dependent trapping/detrapping should be probed by short edge time in the pulse CV measurement, which corresponds to shallower traps (traps at a high energy level within oxide), at least within the timescale considered here. While for the larger value of the edge time, the transient shift of the flat-band voltage shift is attributed to slow electron trapping/detrapping. It is also noted that all the ramp-down traces in Figure 6 are almost overlapped, suggesting that the total trapping level changes little during each charging process (positive bias induced). Similar results have been observed and confirmed by using the conventional CV measurement.

**Figure 7:**
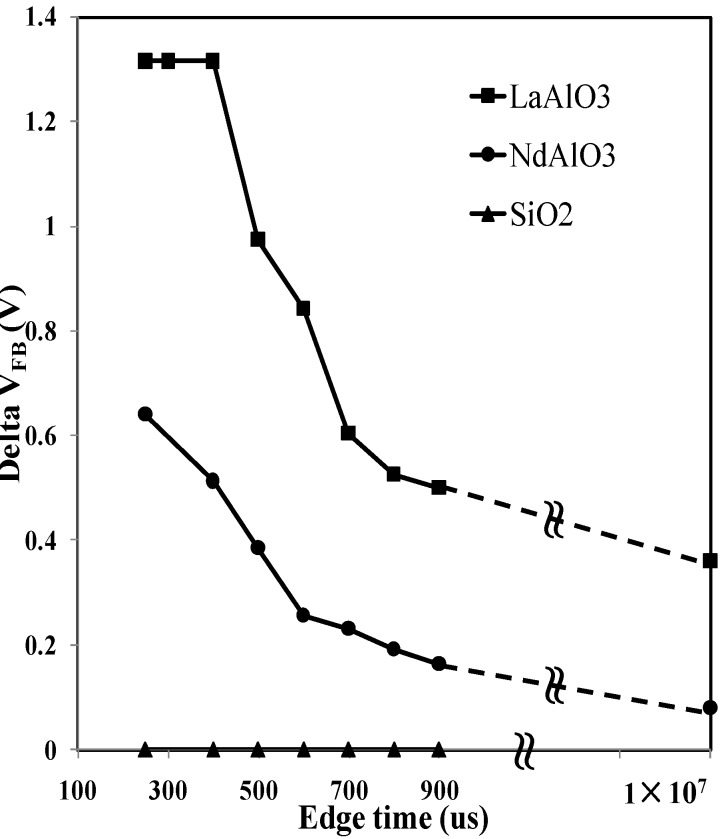
Variation of flat-band voltage (V) with different edging time (μs) of NdAlO_3_, LaAlO_3_ and SiO_2_ samples.

In order to figure out how edge time determines the flat-band voltage shift, two high*-k* materials (LaAlO_3_ and NdAlO_3_) plus a thermal oxide were tested, as shown in Figure 7. By adjusting the edge time, the time-dependent trapping/detrapping was tracked correspondingly. Also, it provided a solution to estimate the entire density of net trapped charges within the oxide under specific edge time. For both high*-k* materials, variations of flat-band voltage were reduced with longer edge time. Due to the hardware limit of the waveform generator, the longest edge time was rated at 900 μs. When the edge time reached 1 × 10^7^ s (10 μs), the pulse CV method was supposed to be a type of conventional CV test. The data obtained via a LCR meter are also shown in Figure 7. It was found that for LaAlO_3_ the variation of the flat-band voltage remained a constant when edge time is less than 400 μs, which means that the entire density of the net trapped charges within the dielectric was measured if edge time is below 400 μs. This is because, once all as-grown electron traps were filled, *V_FB_* would not shift further. The effective trapped charge density now is 1.04 × 10^12^ cm^−2^ (= *C_ox_*∆*V_FB_*/*q*, where ∆*V_FB_* = 1.31 V and *C_ox_* = 1.27 × 10^5^ pF·cm^−2^ is the capacitance density in the accumulation region). ∆*V_FB_* did not return to its fresh value (0.4 V) even under 900 μs of edge time, indicating that some electrons are still trapped within the high-*k* layer. From Figure 7, it was observed that traps of LaAlO_3_ measured by the conventional CV method were less than 31% of the total trapping. Trapping is dominated by the high-*k* layer, which cannot be probed by charge pumping. The charging within 100 s is about 30% of the total, which will be missed if the slow quasi-dc techniques were used. In terms of NdAlO_3_, ∆*V_FB_* was recorded at 0.64 V as the largest value under the edge time of 200 μs, with trap density of 1.14 × 10^12^ cm^−^^2^ (where ∆*V_FB_* = 0.64 V and *C_ox_* = 2.86 × 10^5^ pF·cm^−2^). Similar to LaAlO_3_, the curve trend was also plotted downward and ∆*V_FB_* was close to the fresh value measured using the conventional CV technique. Within the edge time range of 400–700 μs, the slope rate of LaAlO_3_ decreased more significantly than NdAlO_3_, which indicated that trapped electron detrapping was more sensitive to time. It is noted for NdAlO_3_, ∆*V_FB_* did not saturate even at the fastest testing speed of the pulse (200 μs). The entire density of net trapped charges shall be captured using a rapider pulse generator and higher measurement resolution. Concerning thermal oxide, there was no flat-band voltage variation under various edge times, which was also supported by the measurement shown in Figure 4b.

**Figure 8:**
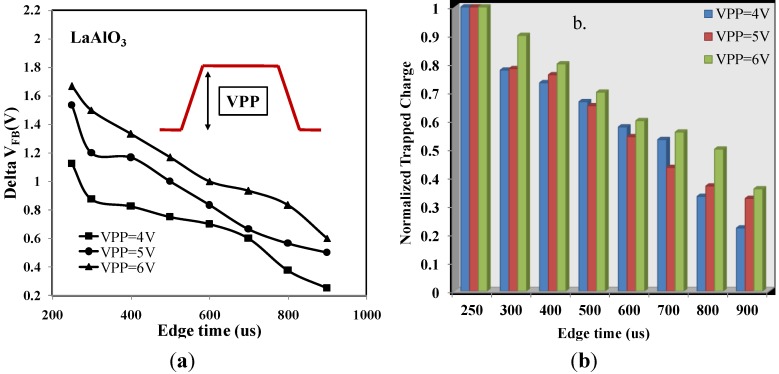
(**a**) Variation of Flat-band Voltage (V) with Different Edging Time (μs) of The LaAlO_3_, Samples under different VPP level. VPP denotes peak to peak voltage of the pulse. (**b**) Normalized Trapped Charge with Different Edging Time (μs).

Finally, the variation of *V_PP_* was investigated for LaAlO_3_, where *V_PP_* denoted the peak to peak voltage of the pulse. When *V_PP_* was 4 V, the pulse started from −2 to 2 V. The definition of *V_PP_* could also be referred in the inset of Figure 8a. The relationship between ∆*V_FB_* and edge time under different *V_PP_* levels was indicated in Figure 8a. It was observed that with stronger *V_PP_* stress, the ∆*V_FB_* remained at a higher level. In all cases, ∆*V_FB_* almost linearly depends on *V_PP_* and reaches a value of 1.67 V at a high voltage (*V_PP_* = 6 V), denoting a rather strong trapping process in the dielectric stacks. The effective trapped charge density is 1.33 × 10^12^ cm^−2^ (where ∆*V_FB_* = 1.67 V and *C_ox_* = 1.27 × 10^5^ pF·cm^−2^), as *V_PP_* equals to 6 V and edge time at 250 μs. Electron traps with deeper energy levels may exist in the bulk of the high-*k* layer, which can only be charged with larger gate bias. Slower ramp rates would cause more detrapping during the CV measurements, while higher ramp rates were limited by the trans-amplifier’s bandwidth. Figure 8b shows the remaining traps during various edge times after taking the pre-existing traps into account. The pre-existing traps are time-independent and also termed as the whole traps. The normalized trapped charge and bar diagram are implemented in Figure 8b.

In general, following the procedure of the pulse CV test, it would be more convincing to characterize the trapping/detrapping mechanism of the electrons/holes in the high*-k* materials, which would be used for the development of the next generation non-volatile memory.

## 4. Conclusions

In this paper, we have introduced a novel electrical characterization for MOS capacitors with high*-k* materials: pulse CV measurement. Different from the conventional CV test, the pulse CV technique could complete the whole test within 1 ms. By using the new technique, the testing time (edge time) and the bias/stress time (width time) could be easily adjusted. Compared to thermal oxides, MOS capacitors with high*-k* materials (like LaAlO_3_ and NdAlO_3_) showed the intrinsic time-dependent trapping/detrapping mechanism via the pulse CV measurement. Various observations concerning the variation of flat-band voltage were discussed accordingly. After understanding the trapping/detrapping mechanism of the high*-k* oxides, the pulse CV technique might be a solid foundation for further exploration into charge-trapping, non-volatile memory based on high-*k* oxides in the coming future.
